# On Hepatic Abscess in India

**Published:** 1837-07

**Authors:** W. Geddes

**Affiliations:** Late Surgeon of the Madras European Regiment


### ON HEPATIC ABSCESS IN INDIA. BY W. GEDDES, ESQ.

[We have received the following letter in reference to an Article in our last Number on the Diseases of India; and, as it contains an explanation of some importance on a point where, it seems, we had mistaken the author's meaning, we have much pleasure in giving it publicity. We may take this opportunity of again calling the attention of our readers to Mr. Geddes's very excellent paper, on a subject of much interest to pathologists in all climates.]

*To the Editors of the British and Foreign Medical Review.*

Gentlemen: In the review of my friend, the late Mr. Twining's Clinical Illustrations, contained in your last Number, allusion has been made, in favorable terms, to an essay of mine on Hepatic Abscess, published in the sixth volume of the Transactions of the Medical and Physical Society of Calcutta. There are many typographical errors, or perhaps some obscurity of expression in that paper, and to these I refer a misconception which seems to have arisen in the Reviewer's mind calculated to shake his confidence, or that of your readers, in the general accuracy of the facts therein detailed. It is stated in the review, as the result of my observations, that of twenty-eight instances wherein abscess of the liver had been found on dissection, pain had existed in thirteen, dysentery in ten, and pyrexia in but five cases. The reviewer seems to have conceived this statement to refer to the presence, or otherwise, of these disorders during the whole progress of the illness; but it is meant to apply, only, to the general aspect under which the hepatic abscess at first showed itself, or, in other words, to the most conspicuous manifestation of disease at the earliest period at which the abscess was considered to have existed. In the further progress of the case to its fatal termination, the predominating symptom continued, as in the commencement, to be pyrexia, pain, or dysenteric disorder; but the first of these affections was distinguished from the others, inasmuch as it almost always accompanied, in some shape or degree, either of the two latter, being, under some varieties, in proportion to their severity; while, as has been alluded to in the review, where the dysenteric symptoms become the predominating affection, there was little pain of side, and, on the other hand, where pain was urgent, there was not much annoyance from affection of the bowels.

I feel much gratified by your calling the attention of your readers to the facts contained in the essay alluded to, presenting as they do a different view of hepatitis when it proves fatal, from what is generally entertained in this country. I am the more anxious, therefore, that nothing of a "startling" nature should appear to have found its way into that production, and this, I trust, will be a sufficient excuse for my troubling you with the above explanation. I am, Gentlemen, yours very obediently,

*Rockhouse, Elgin; May* 13, 1837. W. Geddes,

*Late Surgeon of the Madras European Regiment.*

